# Effects of Combined Supplementation of Conjugated Linoleic Acid, Methionine Chromium, Betaine, and Cysteamine on Meat Tenderness of Rats

**DOI:** 10.1155/2020/5159796

**Published:** 2020-08-07

**Authors:** Lingyuan Yang, Lingmei Zhang, Xingguo Huang, Yulong Yin, Tiejun Li, Jiashun Chen

**Affiliations:** ^1^College of Animal Science and Technology, Hunan Agricultural University, Changsha, Hunan 410128, China; ^2^Hunan Livestock and Poultry Safety Production Collaborative Innovation Center, Changsha, Hunan 410128, China; ^3^CAS Key Laboratory of Agro-Ecological Processes in Subtropical Region, Institute of Subtropical Agriculture, Changsha, Hunan 410125, China

## Abstract

A systemic design was carried out to investigate the optimal combination of BET, Met-Cr, CLA, and CS for improving the meat tenderness in rats. A total of 104 six-week old male Sprague-Dawley rats were randomly assigned to 13 treatments with 4 replicates of 2 rats each. The experiments lasted for 5 weeks. The results showed that inclusion of Met-Cr decreased the contents of intramuscular fat (IMF), fat among muscle cells, and lipid droplets inside muscle cells (*P* < 0.05), and inclusion of CLA or Met-Cr increased the contents of IMF, fat among muscle cells, and lipid droplets inside muscle cells (*P* < 0.05). CS increased the contents of total collagen (TC) and soluble collagen (SC), and CLA decreased the contents of TC and SC (*P* < 0.05). The combination of BET and CLA increased IMF and SC contents and decreased TC contents (*P* < 0.05). The combination of BET and CS could increase fat contents among muscle cells and decrease TC and SC contents (*P* < 0.05). The combination of CLA and Met-Cr decreased IMF contents (*P* < 0.05). The combination of CLA and CS, as well as Met-Cr and CS, decreased fat contents among muscle cells (*P* < 0.05). These combinations may regulate lipogenesis and decrease the deposition of fat in muscles. There existed a significant positive correlation between IMF and SC content, which might indicate that IMF content improves meat's tenderness partly by increasing SC content in muscle.

## 1. Introduction

As living standards have been substantially improved with the rapid growth of the economies of emerging nations such as China and India, their numerous consumers have become more selective of meat quality after they had access to plenty of meat products. Tenderness is an important index of meat quality as it can significantly affect consumer satisfaction [1]. Moderate fat deposition in the muscle could be essential for improving tenderness and flavor [2]. Hence, a new target of livestock workers is to directionally control the redistribution of body fat in the adipose tissue and muscle and then achieve an optimum pattern of distribution. Clenbuterol hydrochloride is a very effective nutrition redistribution agent which can remarkably improve the lean rate in pigs. However, it has been prohibited as a feed additive because its residue in animal products could harmful to the health of consumers. Therefore, various investigations have been conducted to find alternatives to clenbuterol hydrochloride.

Organic chromium (Cr), conjugated linoleic acid (CLA), cysteamine (CS), and betaine (BET) are generally regarded as safe nutritional alternative agents in spite of controversy. BET is an amino acid (trimethylglycine) widely distributed in many organisms and serves as an important intermediate in the catabolism of choline. BET either assists the cellular volume by functioning as an organic osmolyte [3] or provides a methyl for homocysteine to methylate into methionine [4]. Thus, it can partially replace dietary choline and/or methionine and functionally participate in lipid metabolism [5]. CLA is a heterogeneous group of positional and geometrical isomers of linoleic acid, and it is an important bioactive effector to resist tumorigenesis [6, 7] and atherosclerosis [8, 9], regulate immune function [10], and reduce body fat [11–14]. Chromium (Cr), an essential trace element for humans and animals [15], which exists naturally in trivalent and hexavalent states, regulates fat and carbohydrate metabolism presumably by potentiating the action of insulin [16]. Cr has a positive effect on growth performance [17], carcass traits [18], and immunity [19, 20]. CS is a critical metabolite that is biologically generated from cysteine metabolism. It typically serves as a feed additive in animal production, which can stimulate the endocrine system and improve the growth performance and feed efficiency of broilers and pigs [21–23]. CS also reduced back fat thickness and increased the lean percentage of pigs [24]. Furthermore, a previous study has found that dietary BET and CS supplementation had a significant synergistic action on average daily gain (ADG) and feed conversion ratio (FCR), CLA and CS had antagonistic action on ADG, and Met-Cr and CS had a significant synergistic action on FCR in rats [25]. However, most studies only focused on the single effects of one nutritional agent; their combined effects are still largely unknown.

The main objective of the current study is to investigate the potential synergistic or antagonistic effects and to obtain the optimum combination by examining all of the abovementioned meat quality promoters in rats. We assessed different dietary combinations of methionine chromium (Met-Cr), BET, CLA, and CS for rats based on the uniform design, which is one type of experimental design where the representative data are followed with uniform distribution [26, 27]. Moreover, a number of variable levels and experiments can be determined based on the available information. The nonlinearity between responses and factors (i.e., dependent and independent variables) including the coeffects between different factors can be described by the combination of regression analysis. Due to its practicability, uniform design has been widely used not only in physical experiments but also in computer experiments [28]. However, its application in screening the optimal combination of nutrients and/or growth promoters has not been reported. Therefore, another objective of this experiment is to investigate the efficiency of uniform design in exploring the optimal combination that affects meat quality in nutrition research.

## 2. Materials and Methods

### 2.1. Animals, Diets, and Sample Collection

All experimental procedures used throughout this study were approved by the Committee of Animal Ethics at Hunan Agricultural University (permit number: CACAHU 2020-0024). A total of 104 six-week-old male Sprague-Dawley rats were purchased from Dong Chuang Experimental Animal Company (Changsha, Hunan Province, China). All rats were accommodated in a pathogen-free room with a 12 h light/dark cycle (temperature: 23 ± 2°C; relative humidity: 40-70%; the air is changed every 12 h). After a 7 d acclimation period, the rats were randomly assigned to one of thirteen diets. Each dietary group consisted of four cage replicates with two rats in each cage. The diets were based on maize-wheat meal (Table 1) and contained various combinations of BET, CLA, Met-Cr, and CS according to the uniform design (Table 2). The basal diet was formulated to meet or exceed the nutrient requirements for growing rats according to “Laboratory Animals—Mice and Rat Formula Feeds”. Chemical analyses of the diets were performed according to the AOAC (2006) method [29]. All the rats had free access to water and fed on the diets. At the end of the experiments, the rats were sacrificed by decapitation to collect leg muscles and Longissimus dorsi muscle (LM).

### 2.2. Measurements of Intramuscular Fat, Total Collagen, and Soluble Collagen Contents

The muscle sample was shredded and freeze-dried for 70 h. Freeze-dried samples were ground in order to pass through a 40-mesh sieve. The intramuscular fat (IMF) content was analyzed using the classical Soxhlet petroleum-ether extraction.

The contents of total collagen (TC) and soluble collagen (SC) were analyzed, as previously described [30]. Briefly, 1 g dried muscle sample was hydrolyzed in 6 mol/L HCl at 102°C for 24 h to determine the content of total hydroxyproline. 4.5 g of muscle samples were incubated in Ringer's solution (0.7% sodium chloride, 0.03% potassium chloride, and 0.025% calcium chloride) at 77°C for 63 min and centrifuged at 3500 r/min for 6 min, and the supernatant was separated and hydrolyzed in 6 mol/L HCl at 102°C for 24 h to measure the content of soluble hydroxyproline. The contents of total hydroxyproline and soluble hydroxyproline in two acid hydrolysis solutions were determined by spectrophotometry (UV2800, China). The contents of TC and SC were calculated according to the coefficient recommended by Crouse et al. [31]

The fat and lipid droplet contents in muscle cells of LD were determined by the MIASE Medical Image Analysis System (Beihang University, Beijing). The fresh sections of LM medium were fixed with acetone and then dyed with lipid cells, and lipid droplets in muscle cells were observed and photographed under an optical microscope. The fat content among muscle cells was expressed as the ratio of the colored area of fat among muscle cells and the test area, and the lipid droplet content inside muscle cells was expressed as the ratio of the colored area of lipid droplet content inside muscle cells and the test area.

### 2.3. Statistical Analysis

Dietary effects on the different variables were analyzed by one-way ANOVA. Differences between individual means were determined by Fisher's Protected Least Significant Difference (PLSD) multiple-comparison test. Differences were declared significant at *P* < 0.05. The stepwise regression technique was used with DPS software [32] to deal with the quadratic model with IMF, TC, SC, fat, and lipid droplets in muscle cells, respectively.

## 3. Results

### 3.1. Contents of IMF, TC, SC, Fat, and Intramyocellular Lipid Droplets

The contents of IMF, TC, SC, fat, and lipid droplets in muscle cells are shown in Table 3. Due to the lack of uniform design neat comparability, it needs to use regression analysis to get the best combination. Stepwise regression analysis of IMF content has the following regression function:

(1) $$\begin{array}{c} Y=6.7504+0.2923X_{2}-4.6094X_{3}-0.0126X_{4}-0.0151{X_{2}}^{2}+7.9989{X_{3}}^{2}+0.00008484042356{X_{4}}^{2}+0.000010770422950X_{1}X_{2}-0.3588X_{2}X_{3},\\ R=0.9741,\\ F=6.97,\\ P=0.0690,\\ \text{SSE}=0.3029,\\ \text{Ra}=0.9015,\\ {\text{Ra}}^{2}=0.8128, \end{array}$$

where *Y* = IMF content; *X*_1_ = BET dosage; *X*_2_ = CLA dosage; *X*_3_ = Met − Cr dosage; and *X*_4_ = CS dosage, and the same follows.

Partial correlation analysis of BET, CLA, Met-Cr, and CS with IMF showed that CLA was positively related to IMF, but its quadratic dosage was negatively related to IMF. Met-Cr and CS were negatively related to IMF, but their quadratic dosages were positively related to IMF. The interaction between CLA and BET was positively related to IMF, but the interaction between CLA and Met-Cr was negatively related to IMF (Table 4).

Correlation coefficient *R* of the function was 0.9741, and the *P* value was 0.0690. The adjusted correlation coefficient Ra of the function was 0.9015. Although we did not test the result of this combination, the results showed that when the dosages of these nutrition repartitioning were set as 0 mg/kg (as negative control treatment), the predicted IMF content was 6.75%, which was very close to the actual value of 6.50% in the negative control treatment (Table 3). So the regression equation is basically dependable.

An increase of the content of IMF increases meat quality because the IMF content of rats and lean pigs are generally lower. Therefore, the higher the IMF content, the better the pulp. The regression function predicted that when the dosages of Met-Cr, CS, CLA, and BET were 0 mg/g, 0.3 mg/kg, 12.6 mg/g, and 1581.6 mg/kg, respectively, the IMF content would be the highest at 8.20%. This result was better than that of any treatment.

### 3.2. Stepwise Regression Analysis of the Fat Content in Muscle Cells

Stepwise regression analysis of the fat content in muscle cells has the following regression function:

(2) $$\begin{array}{c} Y=0.0041+0.0004X_{2}-0.0093X_{3}-0.00006598859545X_{4}+0.00004493265884{X_{2}}^{2}+0.0120{X_{3}}^{2}+0.0000003580064274{X_{4}}^{2}-0.00000017368648405X_{1}X_{2}+0.000000016036771820X_{1}X_{4}-0.000011598997541X_{2}X_{4}-0.000011456414624X_{3}X_{4}, \end{array}$$

where *Y* is the fat content among muscle cells (area density); *R* = 0.9999; *F* = 49999.93; *P* = 0.0035; SSE = 0.0000; Ra = 0.9999; and Ra^2^ = 0.9999.

Partial correlation analysis of BET, CLA, Met-Cr, and CS with the fat content among muscle cells showed that CLA and its quadratic dosage were positively related to the fat content among muscle cells, and Met-Cr and CS were negatively related to the fat content among muscle cells. In addition, the interactions between BET and CLA, CLA and CS, and Met-Cr and CS were negatively related to the fat content among muscle cells (Table 5).

Correlation coefficient *R* of the function was 0.9999 and *P* value was 0.0035. The adjusted correlation coefficient Ra of the function was 0.9999. Although the combination was not tested, the results showed that when the dosages of the growth promoters were set as 0 mg/kg (as in the negative control treatment), the predicted fat content among muscle cells was 0.004, which was very close to the actual value of 0.003 in the negative control treatment (Table 3). The results indicated that the regression function was reliable.

The regression function predicted that when the dosages of Met-Cr, CLA, CS, and BET were 0 mg/g, 16.0 mg/g, 21.5 mg/kg, and 1597.6 mg/kg, respectively, the content of fat among muscle cells would be the highest at 0.013. This result was better than that of any other treatment.

### 3.3. Stepwise Regression Analysis of Lipid Droplet Content in Muscle Cells

Stepwise regression analysis of lipid droplet content in muscle cells has the following regression function:

(3) $$\begin{array}{c} Y=0.0201-0.0034X_{2}+0.1291X_{3}+0.0012X_{4}-0.1698{X_{3}}^{2}-0.000005782630791{X_{4}}^{2}+0.00000018124939873X_{1}X_{2}-0.000000018509573820X_{1}X_{4}+0.0084X_{2}X_{3}-0.0018X_{3}X_{4}, \end{array}$$

where *Y* is the lipid droplet content in muscle cells (area density); *R* = 0.9967; *F* = 33.48; *P* = 0.0293; SSE = 0.0023; Ra = 0.9817; and Ra^2^ = 0.9637.

Partial correlation analysis of BET, CLA, Met-Cr, and CS with lipid droplet content in muscle cells (Table 6) showed that CLA was negatively related to lipid droplet content in muscle cells, Met-Cr and CS were positively related to lipid droplet content in muscle cells, but their quadratic dosages were negatively related to the lipid droplet content in muscle cells. In addition, the interactions between BET and CLA and between CLA and Met-Cr were positively related to the lipid droplet content in muscle cells. However, the interactions between BET and CS and between Met-Cr and CS were negatively related to lipid droplet content in muscle cells. Path analysis as shown in Table 6 indicated that Met-Cr and CS had a remarkably direct positive effect on the lipid droplet content in muscle cells.

Correlation coefficient *R* of the function was 0.9967, and *P* value was 0.0293. The adjusted correlation coefficient Ra of the function was 0.9817. When the dosages of these nutrition repartitioning were set as 0 mg/kg (as the negative control treatment), the predicted lipid droplet content was 0.020, which was very close to the actual value of 0.019 in the negative control treatment (Table 3).

The regression function predicted that when the dosages of Met-Cr, CLA, CS, and BET were 0 mg/g, 0.001 mg/g, 114.7 mg/kg, and 932.1 mg/kg, respectively, the lipid droplet content would be the highest at 0.082. This result was better than that of any other treatment.

### 3.4. Stepwise Regression Analysis of TC

Stepwise regression analysis of TC has the following regression function:

(4) $$\begin{array}{c} Y=2.7361+0.0001X_{1}+3.0634X_{3}+0.0593X_{4}+0.00000007547320313{X_{1}}^{2}-0.0073{X_{2}}^{2}-0.0002{X_{4}}^{2}-0.000012838559734X_{1}X_{2}-0.000005909215046X_{1}X_{4}-0.0902X_{3}X_{4}, \end{array}$$

where *Y* = TC; *R* = 0.9980; *F* = 55.55; *P* = 0.0178; SSE = 0.0800; Ra = 0.9889; and Ra^2^ = 0.9780.

Partial correlation analysis of BET, CLA, Met-Cr, and CS with the content of TC (Table 7) indicated that BET, Met-Cr, CS, and the quadratic dosage of BET were positively related to TC, but the quadratic dosages of CLA and CS were negatively related to TC. In addition, the interaction between BET and CLA/CS and the interaction between Met-Cr and CS were negatively related to TC.

Correlation coefficient *R* of the function was 0.9980, and *P* value was 0.0178. The adjusted correlation coefficient *R* of the function was 0.9889. The results showed that when the dosages of these nutrition repartitioning were set as 0 mg/kg (as the negative control treatment), the predicted TC content was 2.74 mg/g, which was very close to the actual value of 4.09 mg/g in the negative control treatment (Table 3). The regression function predicted that when the dosages of Met-Cr, CLA, CS, and BET were 0 mg/g, 16.0 mg/g, 21.8 mg/kg, and 1602.9 mg/kg, respectively, the TC content would be the lowest at 1.9 mg/kg.

### 3.5. Stepwise Regression Analysis of SC

Stepwise regression analysis of SC has the following regression function:

(5) $$\begin{array}{c} Y=1.3900+0.0001X_{1}-0.0601X_{2}-0.0048X_{4}+0.000003579188428X_{1}X_{2}-0.0002X_{1}X_{3}-0.0000006335749764X_{1}X_{4}+0.0137X_{2}X_{3}+0.0009X_{2}X_{4}+0.0175X_{3}X_{4}, \end{array}$$

where *Y* = SC; *R* = 0.9995; *F* = 228.13; *P* = 0.0044; SSE = 0.0129; Ra = 0.9973; and Ra^2^ = 0.9946.

Partial correlation analysis of BET, CLA, Met-Cr, and CS with the content of SC indicated that BET, Met-Cr, CS, and the quadratic dosage of BET were positively related to SC, but the quadratic dosages of CLA and CS were negatively related to SC. In addition, the interactions between BET and CLA/CS and between Met-Cr and CS were negatively related to SC (Table 8).

Correlation coefficient *R* of the function was 0.9995, and *P* value was 0.0044. The adjusted correlation coefficient Ra of the function was 0.9973. Although we did not test the result of this combination, when the dosages of these nutrition repartitioning were set as 0 mg/kg (as negative control treatment), the predicted SC content was 1.39 mg/g, which was very close to the actual value of 1.27 mg/g in negative control treatment indicating that the regression function was credible (Table 3).

The regression function predicted that when the dosages of Met-Cr, CS, CLA, and BET were 0 mg/g, 0.1 mg/kg, 2.0 mg/g, and 3200.5 mg/kg, respectively, the SC content would be the highest at 1.6 mg/kg. This result was better than that of any other treatment.

### 3.6. Correlation between IMF, TC, and SC Analysis

Moreover, the Pearson correlation and partial correlation between IMF, TC, and SC contents were also analyzed. As shown in Table 9, there is a light positive correlation existing between IMF and SC (*P* = 0.2149) and a light negative correlation existing between TC and SC (*P* = 0.2001). Furthermore, the reason for showing the partial correlation analysis is that significant correlations were observed between IMF and SC (*P* = 0.0343) and between TC and SC (*P* = 0.0456) (Table 10).

## 4. Discussion

The IMF content is assumed to be positively associated with sensory quality traits, such as the flavor, juiciness, and tenderness of meat, which means a low amount of fat in the muscle results in a less tasty meat. The higher IMF content of beef cuts will boost the flavor ratings and consequently the overall palatability as assessed by consumers since the tenderness of meat is relatively intermediate [33]. It has been shown that BET could increase the IMF content in broilers [34] and pigs [35, 36]. In the present study, the first and quadratic term of BET was not discovered in the regression equations of IMF, in the content of fat among muscle cells, and in lipid droplets in muscle cells; it only appeared as an interaction item. It is possible that the effect of BET on the IMF content was not significant compared with the other nutrient repartitioning agents and had been overshadowed by the effects of the other three agents. However, the specific reasons need to be studied further.

Many studies showed that feeding CLA increased the IMF content in broilers and pigs [37–43], which would be very interesting in terms of meat quality. In this study, CLA increased the IMF content and had a curvilinear effect; however, the IMF content was reduced when the dose of CLA exceeded a certain level. In 2001, Wiegand et al. speculated that if body fat was decreased by CLA supplementation, then less energy would be required to maintain animal growth, thus making them more efficient [11]. These findings are in agreement with results reported by Meadus et al. [37]. Furthermore, the findings of the present study also showed that CLA increased the fat content among muscle cells in a dose-dependent curvilinear pattern, but it linearly reduced the lipid droplets in muscle cells. This indicated that CLA promoted the deposition of fat cells among muscle cells, but it reduced the deposition of lipid droplets in muscle cells. Meadus et al. [37] found that the increased percentage of intramuscular fat by CLA was a result of (1) local stem cells being recruited into an adipocyte lineage by a CLA-activated nuclear transcription factor and (2) increased nutrient supply in and around muscle. However, some other studies showed that CLA had no effect on IMF content [44–46]. Thus, it is still necessary to explore its underlying mechanism in detail.

The existing results on the influence of Cr supplementation on IMF content are inconsistent. It has been reported that supplemental Cr at 0.4 and 2.0 mg/kg from Cr-Pro, Cr-Pic, and CrCl_3_, respectively, had no effects on breast IMF, but significantly increased serum triglyceride regardless of Cr source and Cr concentration in broilers [47]. The reports about the combined impacts of nutrition redistributions on the content of IMF are sparse. It has been shown that the synergistic effects of BET and CLA tended to decrease carcass fat content in growing Iberian pigs [48]. Our study suggested that BET and CLA synergistically increased both the IMF of legs and lipid droplet content inside muscle cells of LD, but they decreased the fat content among muscle cells of LD. CLA alone can increase the content of fat among muscle cells, whereas BET can promote the oxidation of fatty acids in muscle cells. Hence, with the aid of both CLA and BET, the fat among muscle cells may be transferred into muscle cells for fatty acid oxidation. The metabolites of the oxidation may form lipid droplets inside muscle cells. However, no significant synergistic effects of BET and Cr on IMF and lipid droplet content inside muscle cells were observed in our study. To the best of our knowledge, this is the first report on the effects of BET and CS supplementation in combination. Our study demonstrated that the addition of both BET and CS was able to increase fat among muscle cells and reduce lipid droplet content inside muscle cells, and had no influence on IMF of the legs. The combining effects of CLA and Cr have been mainly studied in the models for insulin resistance and obesity [49–51]. It has to be pointed out that no details on IMF were provided in those studies. We showed that CLA and Cr-Met could be used to decrease the IMF of muscles in legs and increase lipid droplet content inside muscle cells of LD; they had no effects on fat among muscle cells of LD. Negative relationships between IMF and insulin sensitivity have been reported, and it has also been reported that the addition of CLA and Cr in high-fat diets improved insulin sensitivity [49], indicating that the synergistic effects may reduce IMF, which was similar to our present results. So far, no reports are available on the application of the combination of CLA and CS. In this study, we showed that no significant effects were observed from the use of CLA and CS on IMF and lipid droplet content inside muscle cells except a reduction in fat among muscle cells. Accordingly, it may be necessary to explore more by optimizing the combination of CLA and Cr so that the synergistic effects can be verified further. The combining effect of Cr with CS has been reported by some researchers [50], but their effects on IMF were not determined in previous studies [52]; their studies showed that supplementation of Cr yeast and CS significantly increased loin muscle area, increased the content of IMF, and improved tenderness. However, there were some discrepancies in their results, which may be due to the differences in the animals used in the test or other factors. As a consequence, further research on the synergistic effects of Cr and Cs is needed.

TC and SC are key factors to muscle tenderness. About 60% of the total muscle protein is composed of myofibrillar proteins, and the rest are mainly sarcoplasmic proteins and stroma proteins. Stroma proteins are largely composed of insoluble connective tissue components (collagen and elastin). Collagen is the main component of connective tissue and its solubility depends on the degree of cross-links. Collagen exists in the whole muscle and in fascicles, together with individual muscle fibers in the form of epimysium, perimysium, and endomysium, forming the connective tissue sheaths which have impacts on the water-binding capacity and tenderness [53]. The more collagen and the lower solubility of the muscle might have led to the higher water-binding capacity and less tenderness [54, 55]. There were no reports on the effects from BET, CLA, Cr-Met, and CS on TC and SC. Our results suggested that BET, Cr-Met, and CS increased whereas CLA decreased the content of TC. Martin et al. in 2008 reported that dietary 1% CLA increased the IMF content in pigs [44]. Our results indicated that the tenderness may not only be from the increase in IMF but also from the decrease in TC. The increase in TC by BET was mainly from the increase in SC. The increase in SC did not reduce the tenderness; therefore, the use of BET resulting in an increase in SC may be beneficial to the tenderness. BET may also increase the diameter of muscle fibers, which may contribute to the decrease in tenderness. The offset from these two consequences might have no significant impact on the tenderness when BET was used [56]. As can be seen from our results, Cr not only increased TC but also decreased SC, while IMF depended on the dosage of Cr. As a consequence, the relationship between Cr and tenderness was not obvious in terms of the content of both TC and SC. Our results suggested that the increase in tenderness was from the increase in IMF instead of the decrease in connective tissue. The increase in TC may lower drip loss and enhance cooking percentage, which is in line with the report that Cr could improve water-binding capacity [57, 58]. TC was increased linearly with CS, and the opposite effect was observed for SC. In terms of connective tissues, CS had negative effects on the tenderness. Some discrepancies were found in the impact of CS on IMF, and the results seem to be related to the animals. Due to no measurement on tenderness, the evaluation of CS on it may be compromised if only considering the amount of connective tissue and IMF in muscle. In addition, some discrepancy existed in the impact of CS on tenderness [59]. However, the mechanism of this phenomenon is not well understood. As for combinations of nutrient repartitioning agents of the investigation, the combination of BET and CLA increased SC but decreased TC. This is the same as the combination of Cr-Met and CS. The combination of CS and CLA could increase SC. All these three combinations had a positive impact on the connective tissues.

## 5. Conclusions

A uniform design was applied in this study, and the optimized combinations and dosages of BET, CLA, Cr-Met, and CS were presented in terms of their effects on the meat quality markers. A decrease in IMF, fat content among muscle cells, and lipid droplet content inside muscle cells was observed with Cr-Met supplementation, while an increase in these markers was observed by both CLA and CS. The combination of BET with CLA increased IMF and SC, and decreased TC, which were beneficial to tenderness. The combination of BET with CS increased the fat content among muscle cells, and decreased TC and SC, which might improve tenderness. The combination of CLA and Cr-Met reduced IMF, and the combinations of CLA and CS and Cr-Met and CS reduced the fat content among muscle cells, which may provide some insights into the study of insulin sensitivity and diabetes. The positive relationship between IMF and SC was noticed as well, which may support the consideration that tenderness resulting from IMF may be due in part to the increase in SC.

## Figures and Tables

**Table 1:** Composition of the basal diet, as-fed basis.

Ingredients (g/kg)		Nutrients^a^	
Corn	270.0	Dry material (g/kg)	852.2
Soy bean meal	170.0	Total energy (MJ/kg)	17.79
Wheat	310.0	Crude protein (g/kg)	206.7
Wheat bran	45.0	Ether extract (g/kg)	92.5
Fish meal	150.0	Crude fiber (g/kg)	28.9
Soy oil	10.0	Ash (g/kg)	59.5
Lard	30.0	Calcium (g/kg)	13.2
Salt	5.0	Phosphorus (g/kg)	6.0
Limestone	10.0		
Premix^b^	10.0		

^a^Analysed values for all dietary components. ^b^Amount provided per kilogram of diet: Cu—13 mg; Fe—270 mg; Mn—64 mg; Zn—70 mg; I—0.8 mg; Se—0.27 mg; Co—0.6 mg; vitamin A—14000 IU; vitamin D3—1500 IU; vitamin E—120 IU; vitamin K3—5 mg; thiamine—13 mg; riboflavin—12 mg; pyridoxine—12 mg; vitamin B12—0.022 mg; biotin—0.2 mg; D-pantothenic acid—24 mg; folic acid—6 mg; niacin—60 mg; choline—1250 mg.

**Table 2:** Combination of BET, CLA, Met-Cr, and CS in each treatment based on uniform form design.

Treatments^a^	Net content of each additive (mg/kg diet)^b^				Sample number
	BET	CLA	Met-Cr	CS	
1	800	1000	0	40	8
2	1600	1000	0.8	20	8
3	3200	0	0.1	0	8
4	400	4000	0.4	0	8
5	1600	8000	0	20	8
6	3200	4000	0.05	160	8
7	0	2000	0.05	10	8
8	6400	16000	0.2	10	8
9	6400	2000	0.4	80	8
10	400	0	0.2	160	8
11	0	16000	0.1	80	8
12	800	8000	0.8	40	8
13	0	0	0	0	8

^a^Treatment 13 was used as a negative control, and it was not included in the uniform design. ^b^BET: betaine; CLA: conjugated linoleic acid; Met-Cr: methionine chromium; CS: cysteamine.

**Table 3:** Effects of BET, CLA, Met-Cr, and CS as well as their interactions on some important meat quality indices.

Treatment	IMF (%)	TC (mg/g)	SC (mg/g)	Fat content among muscle cells	Lipid droplet content in muscle cells
1	6.55^a,b^	4.71^a^	1.24	0.0024^a,b^	0.0559^a^
2	8.00^b^	4.91^a^	1.41	0.0035^a,b^	0.0114^c^
3	6.27^a,b^	4.14^a,b^	1.63	0.0033^a,b^	0.03379^a,b^
4	6.51^a,b^	3.91^a,b^	1.18	0.0044^a,b^	0.0439^a,b,c^
5	7.82^b^	3.32^b^	1.14	0.0055^a^	0.0162^b,c^
6	7.83^b^	3.70^a,b^	1.13	0.0031^a,b^	0.0404^a,b,c^
7	7.30^a,b^	3.35^b^	1.26	0.0038^a,b^	0.0291^a,b,c^
8	6.80^a,b^	3.88^a,b^	1.28	0.0015^b^	0.0372^a,b,c^
9	6.15^a^	4.85^a^	1.42	0.0041^a,b^	0.0409^a,b,c^
10	6.15^a^	3.88^a,b^	1.16	0.0020^b^	0.0281^a,b,c^
11	6.18^a^	3.77^a,b^	1.44	0.0034^a,b^	0.0375^a,b,c^
12	6.93^a,b^	3.75^a,b^	1.62	0.0038^a,b^	0.0244^a,b,c^
13	6.50^a,b^	4.09^a,b^	1.27	0.0031^a,b^	0.0194^b,c^
SEM	0.1882	0.1433	0.0471	0.0003	0.0030

^a,b,c^Means within the same column with different letters differ significantly (*P* < 0.05 for lowercase and *P* < 0.01 for capital letter). IMF: intramuscular fat content; TC: total collagen; SC: soluble collagen; SEM: standard error of means.

**Table 4:** Partial correlation analysis of BET, CLA, Met-Cr, and CS with IMF.

Items	Partial correlation coefficient	*T* value	*P* value
*r*(IMF, *X*_2_)	0.9133	3.8846	0.0178
*r*(IMF, *X*_3_)	-0.8912	3.4031	0.0272
*r*(IMF, *X*_4_)	-0.7079	1.7361	0.1576
*r*(IMF, *X*_2_∗*X*_2_)	-0.8987	3.5493	0.0238
*r*(IMF, *X*_3_∗*X*_3_)	0.9432	4.9151	0.0080
*r*(IMF, *X*_4_∗*X*_4_)	0.7420	1.9171	0.1277
*r*(IMF, *X*_1_∗*X*_2_)	0.7984	2.2965	0.0833
*r*(IMF, *X*_2_∗*X*_3_)	-0.9087	3.7713	0.0196

*X*_1_: BET dosage; *X*_2_: CLA dosage; *X*_3_: Met-Cr dosage; *X*_4_: CS dosage.

**Table 5:** Partial correlation analysis of BET, CLA, Met-Cr, and CS with fat content among muscle cells.

Items	Partial correlation coefficient	*T* value	*P* value
*r*(IMF, *X*_2_)	1	360.7014	0.0001
*r*(IMF, *X*_3_)	-1	216.4755	0.0001
*r*(IMF, *X*_4_)	-1	245.1577	0.0001
*r*(IMF, *X*_2_∗*X*_2_)	1	186.0659	0.0001
*r*(IMF, *X*_3_∗*X*_3_)	1	219.1888	0.0001
*r*(IMF, *X*_4_∗*X*_4_)	1	245.0954	0.0001
*r*(IMF, *X*_1_∗*X*_2_)	-1	345.0792	0.0001
*r*(IMF, *X*_1_∗*X*_4_)	1	326.4331	0.0001
*r*(IMF, *X*_2_∗*X*_4_)	-1	282.3371	0.0001
*r*(IMF, *X*_3_∗*X*_4_)	-0.9995	32.73	0.0009

*X*_1_: BET dosage; *X*_2_: CLA dosage; *X*_3_: Met-Cr dosage; *X*_4_: CS dosage.

**Table 6:** Partial correlation analysis of BET, CLA, Met-Cr, and CS with lipid drop content inside muscle cells.

Items	Partial correlation coefficient	*T* value	*P* value
*r*(IMF, *X*_2_)	-0.9921	11.1837	0.0015
*r*(IMF, *X*_3_)	0.9891	9.4953	0.0025
*r*(IMF, *X*_4_)	0.9923	11.3058	0.0015
*r*(IMF, *X*_3_∗*X*_3_)	-0.9929	11.7877	0.0013
*r*(IMF, *X*_4_∗*X*_4_)	-0.992	11.1408	0.0015
*r*(IMF, *X*_1_∗*X*_2_)	0.9494	4.2744	0.0235
*r*(IMF, *X*_1_∗*X*_4_)	-0.9076	3.0583	0.0551
*r*(IMF, *X*_2_∗*X*_3_)	0.9916	10.8253	0.0017
*r*(IMF, *X*_3_∗*X*_4_)	-0.9923	11.3524	0.0015

*X*_1_: BET dosage; *X*_2_: CLA dosage; *X*_3_: Met-Cr dosage; *X*_4_: CS dosage.

**Table 7:** Partial correlation analysis of BET, CLA, Met-Cr, and CS with TC.

Items	Partial correlation coefficient	*T* value	*P* value
*r*(TC, *X*_1_)	0.7924	1.8370	0.1635
*r*(TC, *X*_3_)	0.9956	14.9653	0.0006
*r*(TC, *X*_4_)	0.9959	15.5295	0.0006
*r*(TC, *X*_1_∗*X*_1_)	0.9855	8.2219	0.0038
*r*(TC, *X*_2_∗*X*_2_)	-0.9939	12.7838	0.0010
*r*(TC, *X*_4_∗*X*_4_)	-0.9943	13.1847	0.0009
*r*(TC, *X*_1_∗*X*_2_)	-0.9701	5.6480	0.0110
*r*(TC, *X*_1_∗*X*_4_)	-0.9952	14.3193	0.0007
*r*(TC, *X*_3_∗*X*_4_)	-0.9951	14.2705	0.0007

*X*_1_: BET dosage; *X*_2_: CLA dosage; *X*_3_: Met-Cr dosage; *X*_4_: CS dosage.

**Table 8:** Partial correlation analysis of BET, CLA, Met-Cr, and CS with SC.

Items	Partial correlation coefficient	*T* value	*P* value
*r*(SC, *X*_1_)	0.9953	14.5274	0.0007
*r*(SC, *X*_2_)	-0.9956	15.0790	0.0006
*r*(SC, *X*_4_)	-0.9864	8.4992	0.0034
*r*(SC, *X*_1_∗*X*_2_)	0.9308	3.6019	0.0367
*r*(SC, *X*_1_∗*X*_3_)	-0.9808	7.1192	0.0057
*r*(SC, *X*_1_∗*X*_4_)	-0.9827	7.5030	0.0049
*r*(SC, *X*_2_∗*X*_3_)	0.7853	1.7935	0.1708
*r*(SC, *X*_2_∗*X*_4_)	0.9962	16.1102	0.0005
*r*(SC, *X*_3_∗*X*_4_)	0.9849	8.0589	0.0040

*X*_1_: BET dosage; *X*_2_: CLA dosage; *X*_3_: Met-Cr dosage; *X*_4_: CS dosage.

**Table 9:** Pearson correlation analysis between contents of IMF, TC, and SC in muscle.

Items	Correlation coefficient	*P* value
IMF-TC	-0.0961	0.3934
IMF-SC	0.2149	0.0540^∗^
TC-SC	0.2001	0.0733^∗^

^∗^ *P* < 0.10 represents a light correlation.

**Table 10:** Partial correlation analysis of IMF, TC, and SC content in muscle.

Items	Correlation coefficient	*P* value
IMF-TC	-0.1454	0.2041
IMF-SC	0.2401	0.0343^∗^
TC-SC	0.2271	0.0456^∗^

^∗^ *P* < 0.05 represents a significant difference.

## Data Availability

The underlying raw data used to support the findings of this study are available from the corresponding author upon request.

## Additional Points

*ORCID*. The ORCID for this study is as follows:https://orcid.org/0000-0002-4474-6803.
